# A practical guideline for intracranial volume estimation in patients with Alzheimer's disease

**DOI:** 10.1186/1471-2105-16-S7-S8

**Published:** 2015-04-23

**Authors:** Saman Sargolzaei, Arman Sargolzaei, Mercedes Cabrerizo, Gang Chen, Mohammed Goryawala, Shirin Noei, Qi Zhou, Ranjan Duara, Warren Barker, Malek Adjouadi

**Affiliations:** 1 Department of Electrical and Computer Engineering, Florida International University, Miami, FL 33174, USA; 2 Scientific and Statistical Computing Core, NIMH/NIH/HHS, USA; 3 Department of Radiology, University of Miami Miller School of Medicine, Miami, USA; 4 Department of Civil and Environmental Engineering, Florida International University, Miami, USA; 5 Wien Center for Alzheimer's Disease and Memory Disorders, Mount Sinai Medical Center, Miami Beach, USA

## Abstract

**Background:**

Intracranial volume (ICV) is an important normalization measure used in morphometric analyses to correct for head size in studies of Alzheimer Disease (AD). Inaccurate ICV estimation could introduce bias in the outcome. The current study provides a decision aid in defining protocols for ICV estimation in patients with Alzheimer disease in terms of sampling frequencies that can be optimally used on the volumetric MRI data, and the type of software most suitable for use in estimating the ICV measure.

**Methods:**

Two groups of 22 subjects are considered, including adult controls (AC) and patients with Alzheimer Disease (AD). Reference measurements were calculated for each subject by manually tracing intracranial cavity by the means of visual inspection. The reliability of reference measurements were assured through intra- and inter- variation analyses. Three publicly well-known software packages (Freesurfer, FSL, and SPM) were examined in their ability to automatically estimate ICV across the groups.

**Results:**

Analysis of the results supported the significant effect of estimation method, gender, cognitive condition of the subject and the interaction among method and cognitive condition factors in the measured ICV. Results on sub-sampling studies with a 95% confidence showed that in order to keep the accuracy of the interleaved slice sampling protocol above 99%, the sampling period cannot exceed 20 millimeters for AC and 15 millimeters for AD. Freesurfer showed promising estimates for both adult groups. However SPM showed more consistency in its ICV estimation over the different phases of the study.

**Conclusions:**

This study emphasized the importance in selecting the appropriate protocol, the choice of the sampling period in the manual estimation of ICV and selection of suitable software for the automated estimation of ICV. The current study serves as an initial framework for establishing an appropriate protocol in both manual and automatic ICV estimations with different subject populations.

## Background

Alzheimer's disease (AD), the most prevalent form of dementia, is affecting the lives of nearly 5.4 million Americans according to the Alzheimer's Association estimates. Regional cerebral atrophy are mostly associated with this neurodegenerative disease in discriminating AD patients from cognitively normal population [1,2]. Magnetic Resonance Imaging (MRI) is a modality often utilized in investigating atrophied regions of cerebrum and in diagnosing prodromal stages of AD. When measuring morphometric features of the brain, normalization is essential in order to account for the different head sizes. Intracranial Volume (ICV) is a standard measure to correct for head size in different brain studies and in particular in AD related literature [3-6].

The ICV measure, sometimes referred to as total intracranial volume (TIV), refers to the estimated volume of the cranial cavity as outlined by the supratentorial dura matter or cerebral contour when dura is not clearly detectable [7]. ICV is often used in studies involved with analysis of the cerebral structure under different imaging modalities, such as Magnetic Resonance (MR) [8,9], MR and Diffusion Tensor Imaging (DTI) [10], MR and Single-photon Emission Computed Tomography (SPECT) [11], Ultrasound [12] and Computed Tomography (CT) [13,14]. ICV consistency during aging [15] makes it a reliable tool for correction of head size variation across subjects in studies that rely on morphological features of the brain. ICV, along with age and gender are reported as covariates to adjust for regression analyses in investigating progressive neurodegenerative brain disorders, such as Alzheimer's disease [4,16-20], aging and cognitive impairment [21]. ICV has also been utilized as an independent voxel based morphometric feature to evaluate age-related changes in the structure of premorbid brain [22-26], determine characterizing atrophy patterns in subjects with mild cognitive impairment (MCI) and Alzheimer's disease (AD) [27,28], delineate structural abnormalities in the white matter (WM) in schizophrenia [29], epilepsy [30-36], and gauge cognitive efficacy [37].

Of the existing protocols for calculating ICV, despite their methodological differences, they can be classified mainly into two broad categories, manual and automated. Manual estimation of ICV involves segmentation of the cranial cavity by hand in every single slice of brain volume. The process of manual segmentation of ICV is a tedious and lengthy process. In order to alleviate this process, different sampling protocols as opposed to considering every slice were suggested and evaluated previously [7]. Calculating ICV following the subsampling protocol [7] reported that no significant loss of measurement reliability (0.999) was observed by segmenting ICV every 10 sagittal slices with 0.938 mm thickness instead of measuring ICV in every single slice. Although subsampling strategies result in significant time saving, this finding was limited to the normal control adult population. Consequently, the first aim of the current study focused on evaluating subsampling protocols for manual estimation of ICV in adult control (AC) and AD population.

Automated approaches for estimating ICV are highly desirable in order to minimize the level of manual intervention required from the human rater in the estimation procedure. Freesurfer [38], FSL [39] and SPM [40] are three widely accepted and well-known software packages in neuroimaging studies, which come with their own routines for estimating ICV. Accuracy of the software packages in estimating ICV has recently been investigated [41]. The main challenge in this reliability assessment is in determining if the estimated ICV through each package is consistent over the variability exhibited with respect to age population, strength of the magnetic field in case of MR based imaging, slice thickness, condition of the population targeted (control or patient) and the type of the neurological disorder [42,43]. Another aim of the current study was placed towards assessing the effect of other factors such as age, gender, filed strength of the MRI on the measured ICV.

Challenges with regards to ICV estimation using different field strengths [44], and in estimating ICV in adult subjects with dementia as the neurological disorder [42,45] have been well addressed in these studies. However these type of evaluations which focused on the use of two software platforms (Freesurfer and SPM), were shown to upwardly bias the ICV for adult subjects [43], and Freesurfer and SPM 5 in the case of subject with dementia [45] have shown an overestimation of the ICV by Freesurfer. Consequently, the third aim of the current work was to provide a reliability assessment of Freesurfer (FS version 5.1.0), FSL (version 5.0) and SPM (version 8) in estimating ICV for the aforementioned two categories of subject groups.

In retrospect, main objectives of the current study are (1) reliability analysis of slice subsampling strategy in cohort of manual ICV estimation in AC and AD populations; (2) Main factors that could affect ICV estimation; and (3) performance evaluation of three commonly used software platforms (Freesurfer, FSL and SPM) for automatically estimating ICV.

## Methods

**Figure 1:**
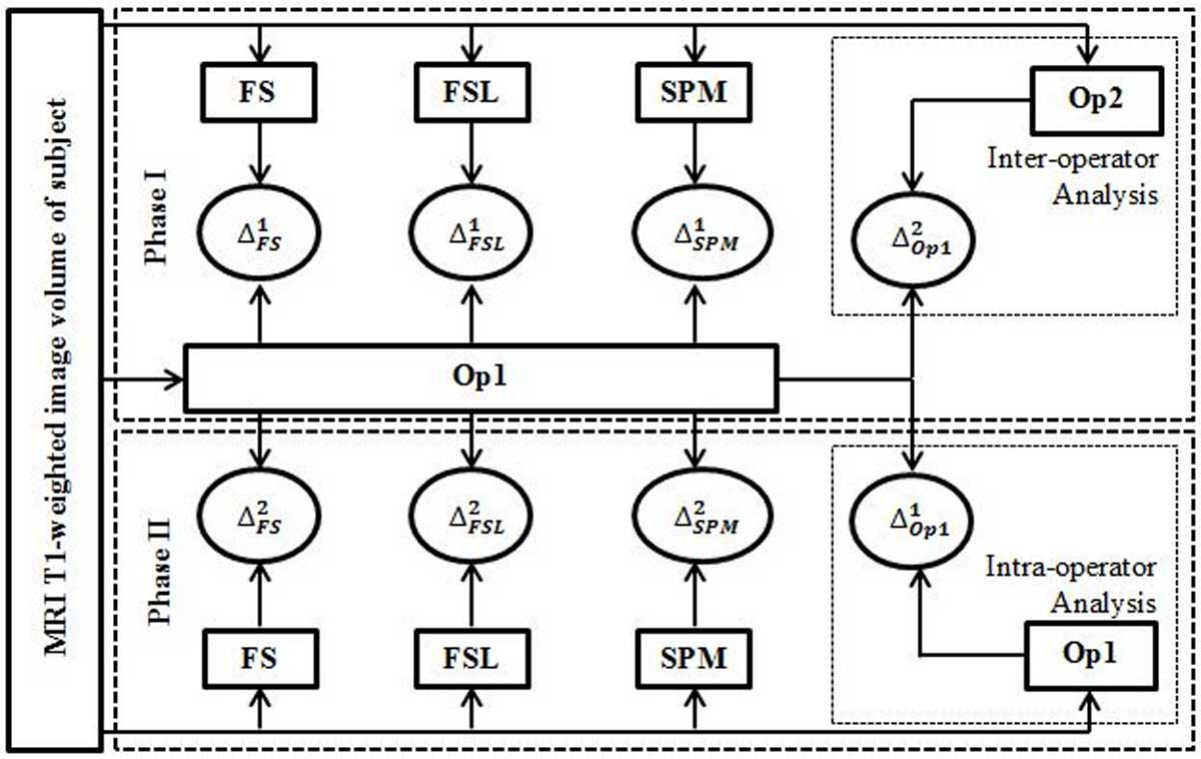
**Overview of the study protocol**. Overview of the study protocol is presented. The study has two phases with phase I implemented two months leading to phase II. Input to both phases of the study is the MRI T1-weighted image volume of the subject. In phase I, Reference manual segmentation of ICV is measured by Operator 1 and the corresponding error of the ICV estimated through automatic software is determined, ${\text{Δ}}_{FS}^{1}$ for Freesurfer, ${\text{Δ}}_{FSL}^{1}$ for FSL and ${\text{Δ}}_{SPM}^{1}$for SPM. Inter-operator variability, ${\text{Δ}}_{Op1}^{2}$, is also calculated in this phase as the error between estimated ICV by Operator 1 and estimated ICV by Operator 2. For the phase II of the study, Intra-operator variability, ${\text{Δ}}_{OP1}^{1}$, is calculated as the error between estimated ICV by Operator 1 in phase I and estimated ICV by the same operator in phase II. The automatic measurements of ICV using Freesurfer, FSL and SPM are repeated and the corresponding errors are calculated as ${\text{Δ}}_{FS}^{2}$ for Freesurfer, ${\text{Δ}}_{FSL}^{2}$ for FSL and ${\text{Δ}}_{SPM}^{2}$ for SPM.

An overview of the study protocol, which includes two phases of analysis, is presented in Figure 1. Phase I of the study, which is two months leading to phase II of the study, involves reference manual measurement of ICV by Operator 1 (**Op1**). The estimated ICV measurements from Freesurfer, FSL and SPM are calculated and contrasted against the reference manual ICV and the errors ${\text{Δ}}_{FS}^{1}$, ${\text{Δ}}_{FSL}^{1}$ and ${\text{Δ}}_{SPM}^{1}$ are calculated for Freesurfer, FSL and SPM, respectively. Second operator (**Op2**) is provided with the same T1-weighted image volume of the subject and inter-operator variability analysis is performed. Intra-operator variability analysis is conducted by re-measuring the ICV by Operator 1 in phase II of the study. Automatic measurements of ICV using FS, FSL and SPM are also repeated to compare the intra-software reproducibility. The second phase of the study was implemented with similar processing power to keep the results unbiased from the potential unbalance processing units in software measurements.

### Subjects and images

Table 1 provides demographic characteristics of the 22 study subjects. All participants are from Wien Center for Alzheimer's Disease and Memory disorders with the Mount Sinai Medical Center, Miami Beach, FL, USA. The study was approved by the local institutional review board (Protocol number: IRB-13-0515) and informed consent forms were provided from the subjects or their legal representatives. Subjects from both groups have taken the Folstein Mini-Mental State Examination [46] with a minimum score of 15 out of 30. AD and AC subjects had a neurological and medical evaluation by a physician acoording to the neuropsychological tests [47]. MRI scans of the brain for Adult population groups, AD and AC, were acquired on a 1.5-T machine (Siemen's Symphony, Iselin, N.J., USA, or General Electric, HDX, Milwaukee, Wisc., USA) using a proprietary 3D-magnetization-prepared rapid-acquisition gradient echo (MPRAGE). Specifications for MPRAGE include coronal sections with a 1.5 mm gap in thickness; section interval, 0.75 mm; TR, 2190 ms; TE, 4.38 ms; TI, 1100 ms; FA, 15°; NEX, 1; matrix, 256 × 256; FOV, 260 mm; bandwidth, 130 Hz/pixel; acquisition time, 9 minutes; phase-encoding direction, right to left.

**Table 1:** Demographic characteristics of study subjects

	AGE	Female/Male
**AD (n = 11)**	81 ± 9.31*	6/5

**AC (n = 11)**	71 ± 6.21	9/2

*Data presented as mean ± standard deviation where applicable.

AC: Adult Control; AD: Adult With Alzheimer's disease.

### Reference ICV estimation

**Figure 2:**
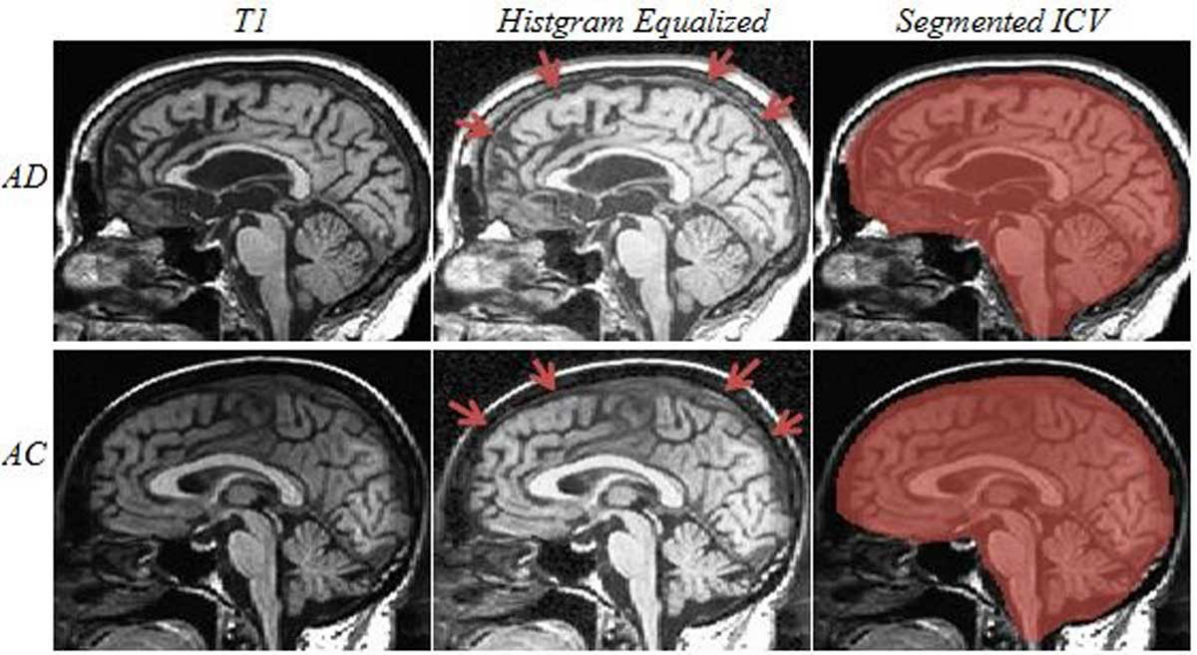
**Reference ICV Segmentation**. Reference ICV segmentation for a sample sagittal slice of randomly selected subject from each group: First row: AD; second row: AC. Left column images are the original T1-weighted image sagittal slice; middle column is the histogram equalized version of the same image with arrows pointing to the boundary of dura which is a landmark in ICV segmentation. Right column is the slice with ICV highlighted pixels.

Reference ICV measurements were performed for all 22 subjects from the two groups by operator 1, Op1, during phase I of the study. Op1 repeated the process of measuring ICV across all subjects during phase II of the study, in order to evaluate intra-operator variability. A second operator, Op2, calculated the ICV of all subjects during phase II of the study to assess the inter-operator variability in calculating ICV. No specific order of subjects/groups was considered by both operators when measuring ICV to lessen the possible learning bias across groups. No time limitation in reference ICV measurement was imposed on the operators, Op1 and Op2. A computer assisted approach, using an AFNI plugin [48], was conducted by the operators to manually draw masks in every slice of the volume and highlighting voxels which belong to ICV. Voxels were included in the ICV mask in each slice by strictly following the protocol from a recent study [43]. The ICV was measured by counting the total number of voxels highlighted as belonging to ICV multiplied by the voxel volume. Figure 2 shows a sample slice of T1-weighetd image volume corresponding to one randomly selected subject from each group with the same slice with ICV highlighted. Their histogram equalized images are shown in the middle column of Figure 2. Arrows are pointing to the visual clarity of dura matter which is considered as a landmark in segmenting ICV from other brain tissues.

### Automatic ICV estimation

During phase I of the study, T1-weighetd image volumes of all subjects in two categories were subjected to ICV estimation through Freesurfer (FS 5.1.0), FSL (FSL 5.0) and Statistical Parametric Mapping (SPM 8). A set of default parameters were chosen when required by the software and no other external interventions were involved. The process of automatic ICV measurement using FS, FSL and SPM was repeated in phase II of the study to evaluate the intra-software variability.

#### FS 5.1.0

T1-weighted image volumes of all subjects were processed through automatic image processing pipeline of Freesurfer software (https://surfer.nmr.mgh.harvard.edu). Estimated ICV reported as part of the statistics file (aseg.stats file) corresponding to the subject, created by the Freesurfer [38], was recorded as the ICV estimation for the subject. ICV estimation by FS is an atlas-based estimation approach [49] which assumes that a correlation exists among ICV of a subject and determinant of the registration matrix used to transform the image from subject space to the atlas.

#### FSL 5.0

ENIGMA protocol (http://enigma.ini.usc.edu) was used to automatically estimate ICV using FSL (http://fsl.fmrib.ox.ac.uk). ICV estimation through FSL [39] following the above mentioned protocol is also an atlas based estimation procedure where the subject image is first linearly aligned to MNI152 standard space. ICV is next calculated by multiplying the inverse of the determinant of the affine matrix by the size of the template brain. The protocol itself corrects for field bias with a two steps BET [50] with an intermediate FAST (automated segmentation tool). The default parameter values of 0.5 and 0 were set respectively for fractional intensity threshold and threshold gradient.

#### SPM 8

VBM toolbox (http://www.fil.ion.ucl.ac.uk/spm) of Statistical Parametric Mapping [51] was used with default parameters to segments the voxels of T1-weighetd brain volume into four classes, namely white matter (WM), gray matter (GM), cerebrospinal fluid (CSF) and other. WM, GM and CSF volumes were summed up to provide an estimate of ICV. To attain the automatic feature of the method, no preprocessing or re-orientation were applied on the T1-weighetd images in advance to estimate the ICV since manual intervention [52]. However visual inspection of the images showed no major misalignment along the commissural line.

### Main factors analysis

To determine the interactions that exist among different factors and covariates, a general linear model was adopted in which ICV was the measured value for each subject. Group (AD, AC), method (Manual, FS, FSL, SPM), sex (M: male; F: female), and age were considered as explanatory variables. The statistical analysis was performed in R [53] using package *afex*.

### Reliability assessment of manual ICV estimation

For each of the AD and AC groups, analysis of intra-operator variation, ${\text{Δ}}_{op1}^{1}$, and inter-operator variation, ${\text{Δ}}_{op1}^{2}$, have been performed through paired t-test and correlation analysis.

### Statistical analysis of sampling based manual ICV estimation

A randomized statistical testing procedure [7] was implemented to measure the accuracy of manual estimate of ICV by changing the sampling period for each group of subjects, AD and AC. Sampling period, *m*, is defined as the number of interleaved slices in tracing ICV across the brain volume, e.g. manual ICV estimation with a sampling period of 2 refers to tracing ICV in only half of the total number of slices. The larger the sampling period chosen, the less amount of time is required for ICV measurement. However, the accuracy of the measurement may drop with different rates for different subject categories. Different sampling periods, beginning from 2, were considered for ICV estimation. At each sampling period, *m*, ICV is calculated from a subset of slices. The first slice of the subset is the slice where the brain tissue appeared for the first time in the sequence of slices. The subsequent slices in the subset were selected every *m *slices from the first slice until the brain tissue is no longer perceived. ICV of the subject was finally calculated as the sum of the traced volumes in the subset multiplied by *m*. The Intra-class correlation coefficient (ICC) among the reference ICV measurement and the estimated ICV at sampling frequency *m *was calculated using two-way random ANOVA test. The process, initiated by randomly selecting the first slice of a given subset and ending with ICC calculation, was repeated five thousand times to create an empirical distribution of ICC's across each group to evaluate the effect of sampling period on the accuracy of the estimated ICV.

### Reliability assessment of automatic ICV estimation tools

For each of the AD and AC groups, three sets of criteria were evaluated to provide a decision aid in choosing automated tool(s) for ICV estimation: (1) Intra-software variations were assessed using paired t-test; (2) Across each subject group, the means of calculated ICV through each automated tool (FS, FSL and SPM) in phase I were tested against the mean of reference ICV measured by Op1, through post hoc t-tests under the general linear model, using R package *phia *(http://CRAN.R-project.org/package=phia); and (3) Mean related percentage of absolute difference (MRPAD) in ICV estimated by each automated tool within each subject group was calculated using equation (1).

$$MRPAD=\frac{1}{n}\sum_{i=1}^{n}\frac{\left|{\text{Δ}}_{aut_{i}}\right|}{ICV_{op1_{i}}}\times 100$$

Where ${\text{Δ}}_{aut_{i}}$ is the error of the specific automated tool in ICV measurement from the reference measurement performed by Op1 in phase I; *aut *represents the automated tool employed: FS, FSL or SPM; and *n *is the number of subjects within the group.

## Results

A summary of intra- and inter-operator variation analysis is presented in Table 2. High correlations (0.999) were observed for intra- and inter-operator measurements of ICV across AD and AC groups. Statistical tests as shown in Table 2 confirm the validity of the manual ICV measurements performed by Op1. ICV measurements performed by Op1 in phase I of the study were then considered as the reference measurements for the rest of the analysis.

**Table 2:** Intra- and inter-operator variation analysis for manual ICV estimation

	*Op1 (Phase I)*	*Op2 (Phase I)*	*Op1 (Phase II)*	${\text{Δ}}_{op1}^{1}$	${\text{Δ}}_{op1}^{2}$	*MRPA*1	*MRPA*1
**AD**	1.4870 ± 0.16418*	1.4870 ± 0.16416	1.4870 ± 0.16418	p = 0.55	p = 0.23	0.002	0.004
**AC**	1.4609 ± 0.14444	1.4610 ± 0.14436	1.4609 ± 0.14446	p = 0.35	p = 0.33	0.002	0.014

*ICV (*mm*^3^) presented as mean ± standard deviation (a factor of 10^6 ^has been taken from all values). *Op1 (Phase I)*: ICV measurements performed by Op1 in phase I of the study; *Op2 (Phase I)*: ICV measurements performed by Op2 in phase I of the study; *Op1 (Phase II)*: ICV measurements performed by Op1 in phase II of the study; ${\text{Δ}}_{op1}^{1}$ is the result for testing the null hypothesis that the ICV measurements performed by Op1 (Phase I) is not significantly different from ICV measurements performed by Op1 (Phase II). ${\text{Δ}}_{op1}^{2}$ is the result for testing the null hypothesis that ICV measurement calculated manually by Op1 (Phase I) is not significantly different from ICV measurements done by Op2 (Phase II). Paired t-test is used. *MRPAD*_1_: Mean related percentage of absolute difference between ICV measurements performed by Op1 in Phase I and Phase II which provides intra-operator error in manual ICV estimation. *MRPAD*_2_: Mean related percentage of absolute difference between ICV measurements performed by Op1 and Op2 in Phase I which is related to inter-operator error; AD: Adult With Alzheimer's Disease subject group; AC: Adult Control subject group.

Group-ID (AC, AD), Sex (Male, Female) and Method (manual, FS, FSL, and SPM) and Age were considered as the main factors of this study. Analyzing their main effects and interactions on measured ICV showed that there is significant overall Group-ID effect (*p *< 0.01), Sex effect (*p *< 0.01) and Method (*p *< 0.01). Furthermore, Interaction between Group-ID and Method factors was the only interaction found to be statistically significant (*p *< 0.01). Table 3 provides all the p-values for the different factors and the interaction between them.

**Table 3:** Main factors analysis and their interactions on the measured ICV

	F	Pr(>F)
Group-ID	4.057815e+00	6.359433e-02
Sex	8.929189e+00	9.777993e-03
Age	1.069814e-01	7.484464e-01
Method	2.096274e+02	2.732017e-25
Group-ID : Sex	8.929189e+00	9.777993e-03
Group-ID : Age	5.299599e-03	9.429964e-01
Sex : Age	2.656019e-01	6.143387e-01
Group-ID : Method	8.024328e+00	2.432221e-04
Sex : Method	8.291057e-01	4.853501e-01
Age : Method	1.981851e-02	9.961500e-01
Group-ID : Sex : Age	1.012292e-03	9.750675e-01
Group-ID : Sex : Method	6.211696e-01	6.052420e-01
Group-ID : Age : Method	2.471838e-01	8.628723e-01
Sex : Age : Method	4.724365e-01	7.031207e-01
Group-ID : Sex : Age : Method	5.051764e-01	6.808114e-01

**Figure 3:**
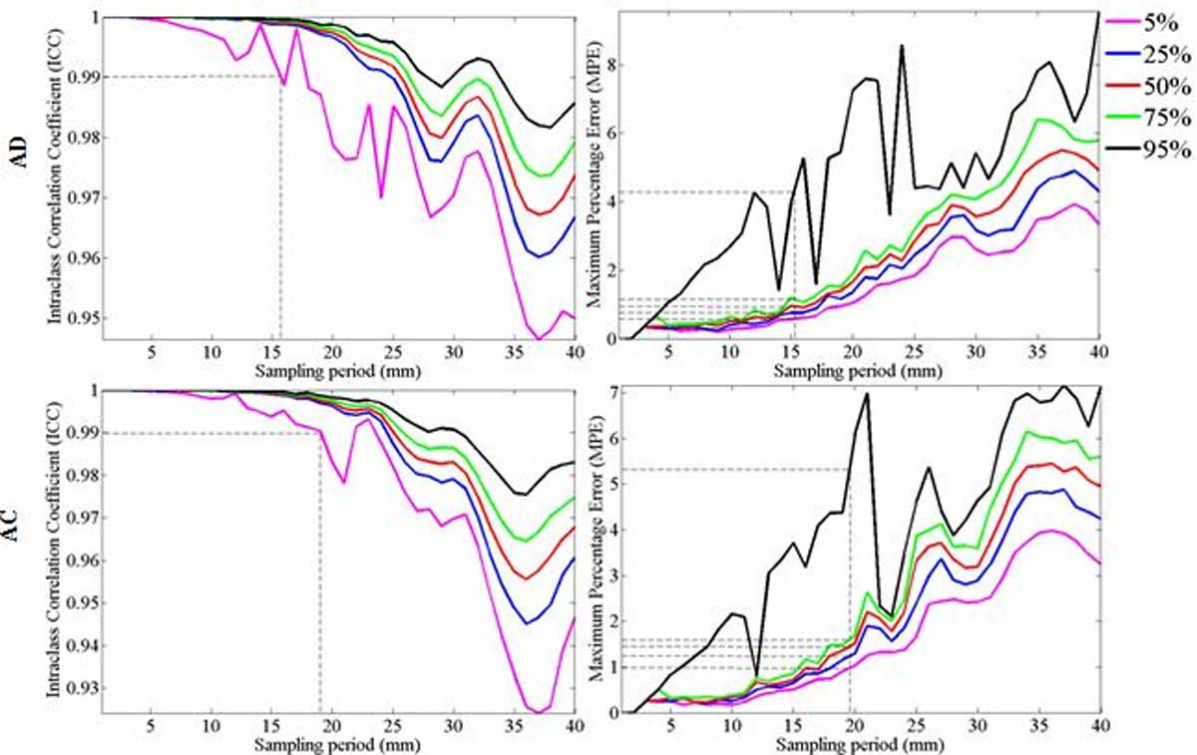
**Reliability assessment of sub-sampling manual ICV estimation**. Left: 5^th^, 25^th^, 50^th^, 75^th^, and 95^th ^percentiles of the empirical distribution of Intra-class Correlation Coefficient (ICC) are plotted for each group contrasting the estimated ICV based on a specific sampling rate with the estimated ICV considering every slice protocol; Right: Maximum Percentage Error (MPE) are given for groups. ICC of 0.99 is highlighted in ICC plots and the corresponding sampling period is found as the point could be considered as a reference to not to lose the accuracy when using subsampling strategies. The sampling period corresponding to the ICC of 0.99 is marked to assess the MPE for that specific sampling period.

Next was to study the effect of the sampling period on the reliability of manual estimation of ICV. For this purpose, random statistical testing procedure explained previously was implemented on ICV measurements performed by Op1 (Phase I) for each subject group. The 5^th^, 25^th^, 50^th^, 75^th^, and 95^th ^percentiles of the empirical distribution of ICC were calculated for each subject group, as shown in Figure 3, to contrast the estimated ICV based on a specific sampling period with the estimated ICV considering the "every slice" protocol. Corresponding maximum percentage errors (MPE) were also given for each sampling period. The figure shows the randomness behaviour of ICC value as the sampling period increases.

**Figure 4:**
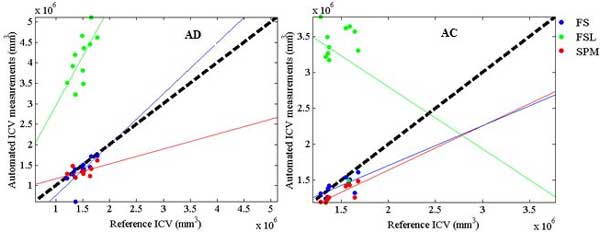
**Reliability assessment of Automatic ICV estimation**. Plot of ICV measurements through automated tools (FS, FSL and SPM) against ICV measurements performed by Op1 in Phase I for each subject group (AD and AC). The bold dashed line defines the reference line, and the solid lines are the regression lines of the ICV estimated by each of the automated tools used.

The sampling periods and corresponding MPE values showed in dashed lines in Figure 3 correspond to the 95% probability of occurrence in keeping the reliability of the estimated ICV measurement above 0.99 based on sub-sampling protocols. Accordingly, a sampling period may not exceed 15.7 *mm *(with MPE ranging from 0.6 to 4.9) for the AD group; and 19 *mm *(with MPE ranging from 0.9 to 4.4) for the AC group. Also by defining the spread of empirical ICC distribution as the difference between the fifth percentile and 95^th ^percentile per sampling period, tight spread were considered as this difference to be lower than or equal with 0.005 [7]. To keep a tighter spread, the sampling period should not exceed 10 mm for AD and 15 mm for AC group. The MPE shows the same random behaviour with an incremental pattern as the sampling period gets higher. The 90% confidence interval of MPE at sampling period of 40 mm is found to be within the range of 3% to 10% for AD and 3.5% to 7% for the AC group. These findings confirm the importance in carefully choosing an appropriate sampling period for the different subject groups when manual estimation of ICV is used. To evaluate the effectiveness of FS, FSL and SPM in the automated ICV estimation process, ICV measurements through the select automated tools are plotted in Figure 4 against the ICV measurements performed by Op1 in phase I. Within each subject group (AD and AC), paired t-test statistics of the difference between each automated tool's ICV estimations and the reference ICV estimations across each group along with correlation coefficient and the corresponding MRPAD values are given in Table 4.

**Table 4:** Software reliability analysis for automated ICV estimation

	AD			AC		
	***p***	***r***	***MRPAD***	***p***	***r***	***MRPAD***

**Op1 vs. FS**	0.1	0.68	9.6	0.13	0.68	5.4
**Op1 vs. FSL**	< 0.0	0.67	177	<0.01	-0.2	127
**Op1 vs. SPM**	< 0.0	0.48	12.5	< 0.01	0.82	10.1

AD: Adult With Alzheimer's Disease subject groups; AC: Adult Control subject groups. *p*: paired t-test p-value; *r*: correlation coefficient; *MRPAD*: Mean Related Percentage of Absolute Difference; p-value less than 0.01 was considered significant.

Intra-software variation analysis is performed across each subject group. Table 5 summarizes the intra-software variation analysis. A retrospective on the results suggest the following findings for each of the study groups:

**Table 5:** Intra-software (Phase I and II) variation analysis for automated ICV estimation

	AD			AC		
	***p***	***r***	***MRPAD***	***p***	***r***	***MRPAD***

**FS**	0.25	0.71	11.5	< 0.0	0.99	1.09
**FSL**	<0.0	0.82	25.4	< 0.0	0.47	33.7
**SPM**	0.49	0.63	5.96	0.1	0.99	0.5

AD: Adult With Alzheimer's Disease subject groups; AC: Adult Control subject groups. *p*: paired t-test p-value; *r*: correlation coefficient; *MRPAD*: Mean Related Percentage of Absolute Difference; p-value less than 0.01 was considered significant.

### AD Subject group

FS showed to be an accurate tool for automatic ICV estimation across AD subject group where a high correlation (0.68) is found among automatic ICV measurements and the reference ICV. MRPAD of FS across AD subject group is found to be 9.6. FS is found to be a reliable tool for AD group as the intra-software variation of FS is found to be non-significant (p = 0.25) and the correlation is ranked second (0.71). SPM is also showed to be a suitable candidate when choosing an automated tool for ICV estimation for the AD group with MRPAD value of 12.5. However, FSL is found to upwardly bias (p < 0.01) the ICV measurements for AD causing a very high MRPAD value (177) as well as a significant variation for its intra-software variation.

### AC Subject group

Both FS and SPM provide competitive results in automatic ICV estimation, MRPAD of 5.4 and 10.1 respectively, which makes them both as good candidates when choosing an automatic tool for ICV estimation across the AC groups. However, the mean of ICV estimated of ICV using SPM is different (p < 0.01) from the one of reference ICV measurement. This results in underestimation of SPM in ICV measurement. SPM shows more reliable (MRPAD value of 0.5) in intra-software variability analysis as compared with FS (MRPAD value of 1.09). FSL is not providing accurate results (correlation coefficient equal with -0.2 and MRPAD of 127) for ICV estimation across AC subject group.

## Discussion

This study was initiated to provide a decision-making process as a guide for estimating ICV either manually or automatically, given the critical importance of ICV as a metric used in brain volumetric studies in reference to AD. The main findings of this study could be summarized in three very important points: (1) The choice of the software should take into consideration whether the population under study is control or AD; (2) the sampling period, in terms of the number of slices that are considered, should be carefully evaluated in terms of the ICC value or accuracy in the ICV estimation, in order to overcome the heavy computational requirements when considering all the slices and the burden imposed in the tediousness of the manual segmentation of ICV; and (3) The analysis of the covariates such as sex, method and group-ID showed that they all have statistically significant effect on the measured ICV. Furthermore the interaction between Group-ID and method was the only interaction between factors that was found to be significant, which supports the importance of choosing a suitable method in calculating ICV with respect to Group-ID.

In assessing the merits of the aforementioned 3 points, four groups of subjects with different neurological conditions, cognitively normal and subjects with Alzheimer's disease were considered: AD: Adult With Alzheimer's Disease; AC: Adult Control. Two operators performed the manual ICV measurements for all the subjects; one of them repeated the measurements in two phases to assess the intra-operator variance. The reliability of reference ICV measurements was assured for intra-operator and inter-operator variations. No statistical significant difference (MRPAD less than 0.01%) was found across the subjects groups considered.

In the case of manual estimation of ICV, the study showed that there is causality between the accuracy and reliability of the measured ICV with respect to the number of slices considered in the segmentation process. The same finding was reported in a previous study using a group of adult control subjects (Eritaia et al., 2000), however the current study reports the existence of similar relationship between the number of slices considered for ICV segmentation and the reliability of the calculated ICV measurement across different subject groups. More importantly, the study also showed the reliability of ICV measurements should be weighed across the different AD and AC groups, and that a set of guidelines should be considered when performing either manual or automatic ICV estimation procedures in terms of both the population under study and the software platform that is used. Consequently, the results shown earlier in Figure 3 could be utilized as a guide in choosing the right sampling period in manual ICV estimation. The current study finds that in order to keep the reliability of the estimated ICV measurement above 0.99 based on sub-sampling protocols; the sampling period may not exceed 15.7 *mm *for AD group; and 19 *mm *for AC group. The sampling periods are given in millimeter unit so to normalize and be capable to apply in other studies.

Furthermore, it was important to set up a decision-making framework in choosing the right software tool in automatic ICV estimation of the two subject groups considered in this study. This was accomplished by evaluating the effectiveness of three widely accepted and well known software packages (FS, FSL and SPM with their default settings) across AD and AC subject groups. The effectiveness of each software was evaluated from two main perspectives: (1) Determining the accuracy of the automatic tool in measuring ICV as compared with reference manual ICV measurement, and (2) Assessing the reliability and consistency of the results for each of the automated software platforms in measuring ICV across subject groups with different neurological conditions.

The results obtained confirmed the hypothesis that the choice of the software should take into consideration whether the population under study is cognitively normal or not, with the knowledge that atlas-based software platforms tend to perform better when dealing with both adult populations. Since the built-in atlas from FS is from normal and Alzheimer subjects [49,54], FS showed excellent results across AD and AC subject groups for this study. On the other hand, SPM-based measurements of ICV showed to be more consistent over different phases of the study across the AC subject group. This could be due to the limited number of defined tissue classes (white matter, gray matter and CSF) which could be discussed more as an advantage when working with subjects with similar conditions to AC subject group. However this could introduce bias when dealing with ICV measurements patients suffering from neurological disorders where brain atrophy is present. Other potential biases in automated ICV estimations are also discussed in related studies [43,44].

## Conclusions

In retrospect, this study emphasizes the importance in selecting the appropriate protocol which should focus on the choice of the sampling period in the manual estimation of ICV and the selection of the most suitable software in the automated estimation of ICV, which are shown to depend largely on the demographics of the targeted population, the imaging parameters of the MR machine, as well as the neurological disorder under study.

The current study serves as an initial framework for establishing an appropriate protocol in both manual and automatic ICV estimations with different subject populations; however it definitely has space for improvement. As ICV has gained its popularity and showed its significance in research area of Alzheimer, this study could serve as an important guide for the researchers of different areas to choose the right approach for a more accurate estimation of ICV.

## Competing interests

The authors declare that they have no competing interests.

## Authors' contributions

Developed and implemented the study algorithm, Designed the experiments: SS, AS, SN, MC, MG, MA. Performed the experiments and designed the study protocols: SS, AS, SN, MG, QZ, RD, WB, MA. Processed the data, prepared the tests, analyzed and interpreted the study results: SS, AS, GC, MA. Performed statistical analyses: SS, GC, MA. Drafting of the manuscript: SS, AS, SN, MG, MC, MA. All authors read and approved the final manuscript.

## Declarations

The publication costs for this article were partially funded by the FIU Open Access Publishing Initiative.

This article has been published as part of *BMC Bioinformatics *Volume 16 Supplement 7, 2015: Selected articles from The 11th Annual Biotechnology and Bioinformatics Symposium (BIOT-2014): Bioinformatics. The full contents of the supplement are available online at http://www.biomedcentral.com/bmcbioinformatics/supplements/16/S7.
